# 3D-Spatial encoding with permanent magnets for ultra-low field magnetic resonance imaging

**DOI:** 10.1038/s41598-018-37953-1

**Published:** 2019-02-06

**Authors:** Michael W. Vogel, Ruben Pellicer Guridi, Jiasheng Su, Viktor Vegh, David C. Reutens

**Affiliations:** Centre for Advanced Imaging, University of Queensland, Brisbane, Queensland Australia

## Abstract

We describe with a theoretical and numerical analysis the use of small permanent magnets moving along prescribed helical paths for 3D spatial encoding and imaging without sample adjustment in ultra-low field magnetic resonance imaging (ULF-MRI). With our developed method the optimal magnet path and orientation for a given encoding magnet number and instrument architecture can be determined. As a proof-of-concept, we studied simple helical magnet paths and lengths for one and two encoding magnets to evaluate the imaging efficiency for a mechanically operated ULF-MRI instrument with permanent magnets. We demonstrate that a single encoding magnet moving around the sample in a single revolution suffices for the generation of a 3D image by back projection.

## Introduction

The conventional setup of magnetic resonance imaging (MRI) or nuclear magnetic resonance (NMR) instruments comprises a static magnet field to magnetize the sample; a system of transmitter and receiver coils to generate and detect a sample signal; and a coil system to encode spatial information for image generation^1^. Image quality depends mainly on signal-to-noise ratio (SNR) which increases with the magnitude and homogeneity of the main magnetic field (commonly referred to as B_0_). This has been the primary motivation for increases in magnetic field strength in MRI and NMR instruments^2,3^. However, superconducting magnets and advanced cryogenics are required to generate such high magnetic field strength, increasing the bulk and cost of purchase, operation and maintenance of these instruments.

The last decade has seen the development of ultra-low magnetic field (ULF) NMR/MRI instruments with main magnetic fields below 10 mT^4–12^. The low field strength at ULF enables novel applications including imaging in the presence of metal offering important future applications for example in trauma, disaster and battlefield imaging^7^. Superconducting technology is not required for magnetic field generation, enabling portable, low power operation. Moreover, the Larmor frequency, related to the magnetic field strength by the Larmor relation *ω*_*L*_ = γ · |B|, with γ = 42.576 MHz/T^1^ being the gyromagnetic ratio for protons (^1^H), is close to the Eigenfrequencies of a number of molecular and physiological processes^7^. This opens the opportunity to new imaging methods sensitized, for instance, for slow diffusion processes, molecular tumbling and protein folding which are difficult to observe at high field^7,9^. Like in the high field regime ULF-MRI/NMR is based on the phenomena of magnetic resonance, however, signal generation and operation differs. Prior to any measurement a pulsed magnetic field which is approximately three orders of magnitude higher (~0.05–0.1 T) than the Earth’s field is applied (also known as pre-polarization) to enhance net sample magnetization according to Curie’s law^4,7,13,14^. Instead of radiofrequency (RF) pulses, signals in ULF-MRI/NMR are generated by the switch to a second magnetic field, the measurement field, oriented perpendicular to the pre-polarization field.

We previously described the use of adjustable small permanent magnet arrays (SPMA’s) that exploit the advantages of Halbach arrays to generate and dynamically control the magnetic fields in ULF-MRI/NMR^4^. Cooley at el. harnessed the intrinsic static field inhomogeneity of a Halbach array for spatial encoding and back projection, a method applied in early conventional MRI^15^, was employed for image reconstruction^15,16^. For 2D spatial encoding, the Halbach array was rotated about the sample and RF pulses were required for 3D imaging^6,16^. Bluemler extended this approach, proposing nested Halbach arrays to generate 2D linear gradients by superposition of two quadrupole fields to avoid complex image reconstruction methods^17^.

**Figure 1:**
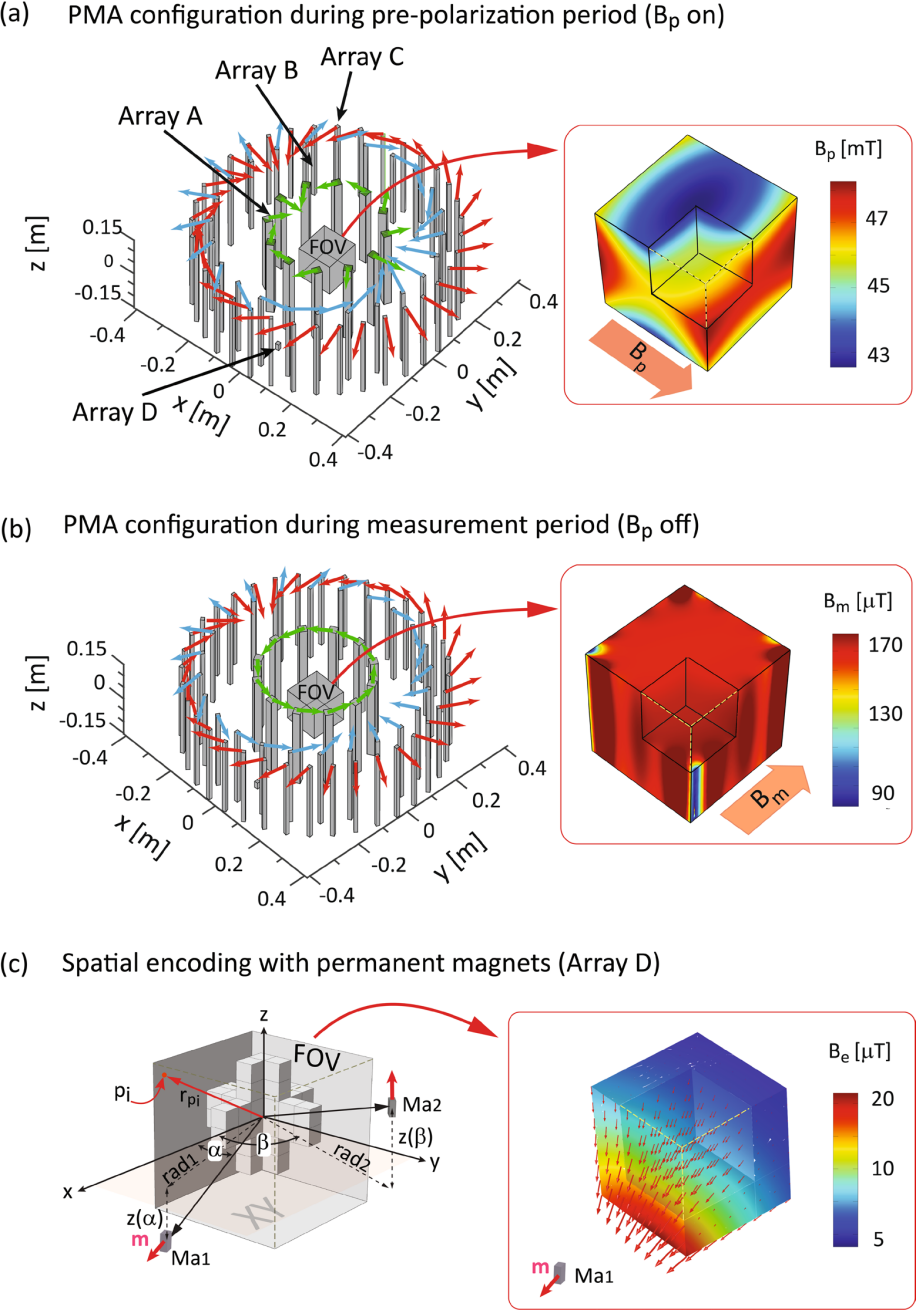
PMA design for the ULF-MRI developed at the Centre for Advanced Imaging (CAI). It comprises a switchable Array A with 12 magnets for sample pre-polarization field **B**_p_, Arrays B and C with 24 magnets and 36 magnets, respectively, and Array D shown with one encoding magnet. With the chosen design parameters, described in the methods section, **B**_p_ ≈ 48 mT parallel to the x-axis and **B**_**m**_ ≈ 140 µT aligned with the y-axis. The magnetisation directions are indicated by green (Array A), blue (Array B) and red (Array C) arrows for each magnet. The insets show the simulated fields as surface plots (COMSOL colour scheme Rainbow) on the cubic FOV, located at the centre of the arrays, with a section removed to view the fields within the FOV. (**a**) Array A with the Halbach magnetization pattern. The **B**_p_ distribution illustrated in the inset has the typical field characteristics of a cylindrical dipole Halbach array. (**b**) Array A with the tangential magnetisation pattern (**B**_p_ = off), with **B**_m_ shown in the inset. (**c**) Detail of FOV with the 3D cross-shaped sample used for this study. For illustration purposes the front section of the sample has been removed. Shown are two small encoding magnets Ma1 and Ma2 with position parameters used in Equations 5 and 6. The inset shows the magnetic field **B**_e_ generated by Ma1 with magnetisation m at an arbitrary location. The red arrows indicate the magnetic field orientation at discrete locations within the FOV; their length indicates the local field strength.

Here, we report on the application of dynamically adjustable permanent magnets moving along prescribed paths to generate 3D spatial encoding field configurations for ULF-MRI without sample motion. As a proof-of-principle, we developed a semi-analytical and numerical approach to determine the most suitable magnet location and orientation to generate 3D encoding fields and demonstrate its applicability for an in-house designed cylindrical ULF-MRI instrument shown in Fig. 1. With our approach, 3D spatial encoding can be achieved without additional RF pulses or relative sample motion to the instrument.

## Methods

### The ULF-MRI instrument model

Figure 1 illustrates the schematic design of the ULF-MRI instrument with permanent magnet arrays (PMA’s) developed at the Centre for Advanced Imaging (CAI) at The University of Queensland. It comprises four concentrically arranged cylindrical arrays: *Array A* with 12 individually rotatable magnets (green arrows) for switching the *pre-polarization field* **B**_p_ to generate sample magnetization; *Arrays B* and *C* with 24 (blue arrows) and 36 (red arrows) magnets, respectively, for generating the *measurement field* **B**_m_^4^; and the *Encoding Array D* with two permanent magnets that creates 3D spatial non-linear *encoding fields* B_e_ for image acquisition. The arrows on each magnet indicates the magnetization direction of each. **B**_p_ is aligned with the x-axis and switched on by individual magnet rotation to form the Halbach magnetization pattern (Fig. 1a) and switched off when the magnets form the tangential magnetization pattern (Fig. 1b). **B**_m_ is generated along the y-axis by Arrays B and C which both have a Halbach magnetization pattern. Superposed magnetic fields cancel within the field of view (FOV) when the magnet orientations in Arrays B and C are in opposing directions. Rotation of the arrays in opposite directions about the z-axis with a relative rotation angle between the arrays of *δ*_*BC*_ sets the magnitude of **B**_m_.

We chose optimized air-core magnetometers for ULF-MRI signal detection^18^. Signal detection with a single surface coil (diameter 120 mm) placed 3 mm away from the sample, and oriented perpendicular to **B**_p_, **B**_m_, and the sample surface was used in the simulation.

### Simulation environment

The intricate setup of the ULF instrumentation with permanent magnets precludes a rigorous theoretical analysis of the magnetic field generation. Instead, full scaled 3D computational models were created in COMSOL^©^, a commercial finite element method (FEM) based simulation platform (version 5.0, modules AC/DC and Magneto-static, COMSOL Inc., Burlington, MA 01803, USA) was employed for numerical analysis to evaluate the static and dynamic magnetic fields. Each model was discretized in 3D-tetrahedral meshes using predetermined and optimized mesh distributions implemented. Near the magnet surfaces and within the FOV the mesh density was manually increased to achieve sub-millimeter spatial resolution to ensure high accuracy and convergence of the results. Typically, the number of tetrahedral elements ranged between 27–28 million with each simulation taking 12–24 hours. A computational cylindrical domain size (diameter 2.175 m, height 1.17 m) with predetermined magnetic shielding boundary conditions (implemented in our previous studies^4,13^) was set to be sufficiently large to minimize numerical errors due to domain discontinuities.

The array diameters were set as follows: for Array A 0.36 m, Array B 0.7 m, and Array C 0.81 m. The array height was set to 0.3 m. Two small ferrite magnets, *Ma*_1_ and *Ma*_2_ (each 6 × 12 × 25 mm) located at a transversal distance *rad1* and *rad2* (Fig. 1c) were implemented in Array D. The remanent magnetization **B**_r_ of the magnets were set to 1.45 T for Array A, corresponding to Neodymium (class N52), while for the other magnets **B**_r_ = 0.4 T, corresponding to commercially available ferrite magnets. The relative permeability for all magnets was set to µ_r_ = 1.05^19^ and for the surrounding air it was µ_r_ = 1. With the design and magnet parameters chosen **B**_p_ ≈ 48 mT and with *δ*_*BC*_ = 5° **B**_m_ ≈ 140 µT, corresponding to a Larmor frequency ≈ 6 kHz for protons (^1^H). The magnitude of **B**_e_ generally ranges from 1–10 µT within the FOV (Fig. 1c), corresponding to a frequency spread of 43–430 Hz. This is comparable to **B**_m_ and well within the bandwidth of our recently developed highly sensitive coil-based magnetometers^18^.

A 3D cubic cross-shaped digital phantom (Fig. 1c) with an arbitrary spin density of 5 compared to a background spin density of 0 was modelled using typical soft tissue relaxation times at ultra-low field of T1 = 100 ms and T2 = 80 ms^20^. The sample was placed within a FOV, represented by equally distributed 8 × 8 × 8 measuring points *p*_*i*_ with overall dimensions 0.12 m × 0.12 m × 0.12 m. At each measuring point the magnetic fields were evaluated in COMSOL and imported into in-house programs, developed in MATLAB (MathWorks^©^, Natick, MA, USA), for virtual signal generation image reconstruction, and to determine optimal magnet location and orientation for the given instrument architecture. The COMSOL simulations were carried out using an x64-based 16 core PC with 128 GB of RAM, while the MATLAB simulations were run on an x64-based 8 core PCs (DELL^©^ Optiplex 9020) with 32 GB of RAM.

### Image acquisition with back projection

#### The encoding matrix

Since the magnetic fields produced by **B**_m_ and **B**_e_ are non-linear, Fourier transform-based image reconstruction methods used in standard MRI are not suitable. This is because non-equidistant k-space filling due to non-linearity, if uncorrected, results in distortions and inhomogeneous image resolution. Instead, we have applied a back projection-based image reconstruction method using the following general relation between the signal at time *t*, the sample magnetization **m(q)** at spatial locations **q** and an encoding matrix **E**_enc_:

1 $${\bf{S}}(t)={{\bf{E}}}_{{\bf{e}}{\bf{n}}{\bf{c}}}({\bf{q}},t)\cdot {\bf{m}}({\bf{q}})$$

Each matrix element of **E**_enc_ describes the time-dependent phase accumulation of the precessing magnetization vectors, which depends on the local magnetic field strength, assumed to be generated by the PMA only, and the acquisition time^6,16^.

#### Simulation of signal generation

We simulated a simple pulse-and-collect experiment with a measurement divided into pre-polarisation, transition and signal detection periods^4,13^. During pre-polarisation the net sample magnetization **M** is generated with **B**_p_. In the transition period **B**_p_ is switched off rapidly or non-adiabatically to avoid **M** following the resultant field^7,21^. Hence, additional RF pulses are not required to flip **M** away from **B**_m_ for MR signal generation. After the transition period, the decaying signal is measured in the presence of **B**_m_ and **B**_e_. For the simulation, it was assumed that **B**_m_ was present throughout the experiment since its significantly lower magnitude does not interfere with **B**_p_. After each measurement period, the encoding magnets move to the next location along a prescribed path to generate **B**_e_. Since it is assumed that their positions are changed during pre-polarization, **B**_e_ can be included as an additional non-linear static field to **B**_m_. According to Equation 1, an encoding matrix **E**_enc_, sized *Q* × *Q*, is required to image a sample composed of *Q* voxels with *Q* different time signals *S(t)*. Hence, with signal acquisitions at *N* time points per measurement, *Q*/*N* different encoding fields are required. We assume that each signal acquisition starts after the transition period at *t*_*s*_ = 10 ms with a sampling interval of 100 µs. The short time windows take into consideration the rapid T1 and T2 relaxation times of tissue at ULF (<100 ms), weak signal amplitude, spin decoherence and other T2* effects caused by the non-linear encoding fields. The accumulated phase is evaluated numerically and included in the encoding matrix. After each measurement period **B**_p_ must be reapplied, since the net sample magnetization **M** has decayed in magnitude with an orientation determined by **B**_m_ and **B**_e_.

The temporal evolution of **M** is described by Bloch’s equation^1,14^ while the resulting magnetic field change induces a voltage in a single receiver coil. For accurate signal representation, a realistic sensitivity profile is implemented based on the principle of reciprocity^22,23^. The resultant MR signal, generated by the precession of protons (^1^H), is calculated by the superposition of signals originating from the discrete measurement locations *p*_*i*_. The effects of spin-spin interactions on the signal which are prominent at ULF were assumed to be included in the relaxation times T1 and T2. It should be noted that the signal originates from the entire sample since no planar slice selections were implemented.

At discrete sample locations *q* with magnetisation *m*_*q*_, the signal *S(t)* acquired for the *p*^*th*^ encoding field configuration at time *t* after pre-polarisation is described as:

2 $${{\rm{S}}}_{p}(t)=\sum _{q=1}^{Q}{m}_{q}{e}^{-j{\omega }_{qp}t}$$

where *ω*_*p*,*q*_ (*p* = 1, 2. *P*, *q* = 1, 2 … *Q*) is the Larmor frequency for a voxel corresponding to location *q* and encoding field configuration *p*. The initial phase for each voxel is assumed to be 0. Using the Bloch equations, Equation 2 can be recast as:

3 $$(\begin{array}{c}\begin{array}{c}{{\rm{S}}}_{1}(t)\\ \begin{array}{c}{{\rm{S}}}_{2}(t)\\ :\end{array}\end{array}\\ {{\rm{S}}}_{p}(t)\end{array})=(\begin{array}{cc}\begin{array}{cc}{e}^{-j\gamma {B}_{11}t} & {e}^{-j\gamma {B}_{21}t}\\ {e}^{-j\gamma {B}_{12}t} & {e}^{-j\gamma {B}_{22}t}\end{array} & \begin{array}{cc}\ldots . & {e}^{-j\gamma {B}_{q1}t}\\ \ldots . & {e}^{-j\gamma {B}_{q2}t}\end{array}\\ \begin{array}{cc}: & :\\ {e}^{-j\gamma {B}_{1p}t} & {e}^{-j\gamma {B}_{2p}t}\end{array} & \begin{array}{cc}\ldots . & :\\ \ldots . & {e}^{-j\gamma {B}_{qp}t}\end{array}\end{array})(\begin{array}{c}\begin{array}{c}{m}_{1}\\ {m}_{2}\end{array}\\ :\\ {m}_{q}\end{array})\equiv {{\boldsymbol{E}}}_{enc}\cdot {\boldsymbol{m}}$$

### Image reconstruction and encoding field configuration

Inverting **E**_enc_ is the most straightforward method to retrieve the image information from Equation 3. This, however, requires **E**_enc_ to be a square matrix. Matrix inversion using standard methods such as Gauss-Jordan elimination or LU decomposition is problematic for large matrix sizes required by high image resolutions or by acquisitions using multiple receiver coils^24,25^.

Figure 1c shows the parameters used to calculate the local magnetic flux density **B** generated by one magnet dipole with magnetization m. The dipole approximation is applicable since the encoding magnets are much smaller than the distance to the sample. The far field approximation yields the magnetic field of the dipole^26^:

4 $${\boldsymbol{B}}=\frac{{\mu }_{0}}{4\pi }(\frac{3{\boldsymbol{r}}({\boldsymbol{m}}\cdot {\boldsymbol{r}})}{{|{\boldsymbol{r}}|}^{5}}-\frac{{\boldsymbol{m}}}{{|{\boldsymbol{r}}|}^{3}})$$

with **r** = **r**_*pi*_ − **r**_*dp*_ being the vector connecting the dipole (magnet) location, **r**_*dp*_, with the point of measurement, **r**_*pi*_. *p*_*i*_ is the *i*-*th* location within the discretised FOV and **m** is the local magnetisation at this point. According to the superposition principle the resultant magnetic field is the sum of the fields generated by *n* encoding magnets and is substituted into Equation 3 to generate the encoding matrix.

We aimed to maximise the rank of the encoding matrix, which reflects the number of linearly independent rows. We also aimed for a low condition number which corresponds to a well-conditioned problem (e.g. matrix data) and in the setting of image reconstruction leads to higher encoding efficiency and lower loss of precision. A high condition number indicates an undesired ill-conditioned problem or a high loss of precision.

Equation 4 permits the implementation of any magnet path by the suitable choice of r_dp_. For the sake of brevity we examined magnet paths that were feasible for the ULF-MRI instrument design (Fig. 1). Two encoding magnets, *Ma1* and *Ma2* moving in cylindrical helical paths around the sample were simulated. The helical path for *Ma*_1_ is described by:

5 $${x}_{Ma1}=Ra{d}_{1}\cdot cos(\alpha ),{y}_{Ma1}=Ra{d}_{1}\cdot sin(\alpha ),{z}_{Ma1}(\alpha )=A{\alpha }^{2}+B\alpha +C$$

where *α* denotes the transverse (*xy*-plane) rotation angle of the cylinder (Fig. 1c) with respect to the *x*-axis. The coefficients *A*, *B* and *C* are given by

6 $$[\begin{array}{c}A\\ B\\ C\end{array}]={(\begin{array}{ccc}{\alpha }_{1}^{2} & {\alpha }_{1} & 1\\ {\alpha }_{2}^{2} & {\alpha }_{2} & 1\\ {\alpha }_{3}^{2} & {\alpha }_{3} & 1\end{array})}^{-1}[\begin{array}{c}z({\alpha }_{1})\\ z({\alpha }_{2})\\ z({\alpha }_{3})\end{array}],$$

*α*_1_ are *α*_3_ are the *starting* and *end* angles and *α*_2_ is the intermediate angular position. *α*_2_ is defined where the helical curves intersect with the transverse plane at *z* = 0. If *α*_2_ = (*α*_3_ − *α*_1_)/2, the height variation *z*(*α*) is a linear function of *α*. The equations describing the helical path of *Ma*_2_ are obtained by substituting *Rad*_2_ for *Rad*_1_ and *β* for *α* in Equations 5.

**Figure 2:**
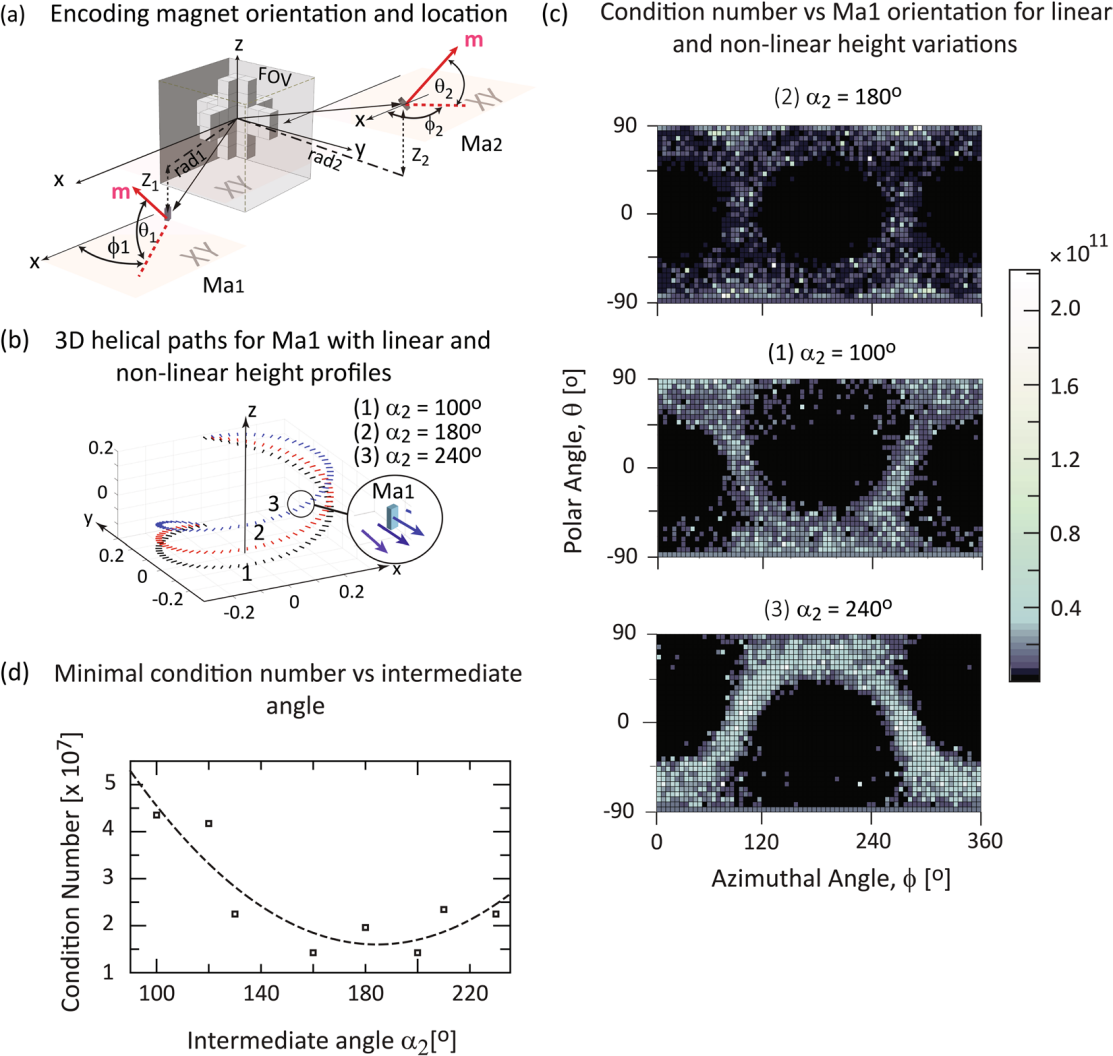
3D encoding magnet paths and corresponding condition number exemplified for one encoding magnet Ma1. (**a**) 3D view show the position angles for Ma1 and Ma2. The magnet orientation **m** is described by the polar angle θ with respect to the x-axis and azimuthal angle ϕ to the xy-plane. (**b**) For Ma1 three helical paths are shown with linear and non-linear height variation z_1_(α). The height varies from z_1_(α_1_) = −0.15 m to z_1_(α_3_) = 0.15 m. Each line segment corresponds to one encoding step location and magnet orientation for Ma1 (see inset), shown here for θ = 0° and ϕ = 0°. α varies from α_1_ = 0° (initial angle) to α_3_ = 360° (final angle), equivalent to one revolution. z_1_(α) varies linearly if the intermediate angle α_2_ = 180° (red path 2) and quadratically if α_2_ = 100° (black path 1) and α_2_ = 240° (blue path 3). (**c**) Condition number vs possible Ma1 orientation for the helical paths shown in (**b**). (**d**) Minimum condition vs intermediate angle α_2_. (**c**,**d**) confirm that the optimal height variation for Ma1 is nearly linear (α_2_ ≈ 180°) with optimal orientation θ ≈ 0° and ϕ ≈ 0°.

**Figure 3:**
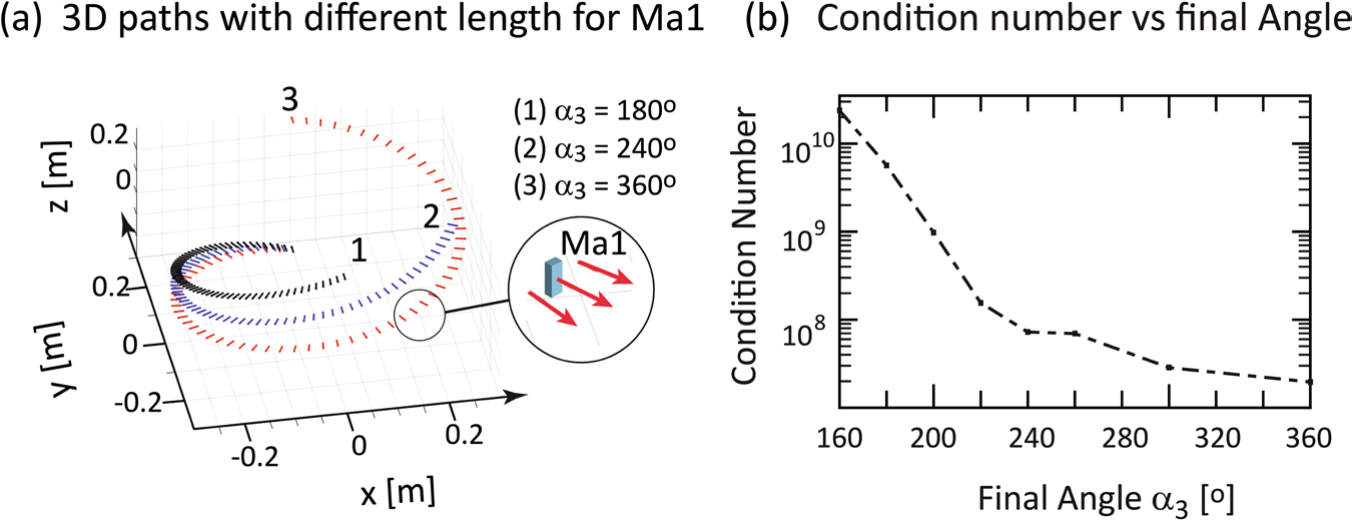
Minimum condition number vs encoding Ma1 path length for linear height variation. (**a**) 3D helical paths with different lengths are shown for final angles α_3_ = 360° (red path 3), α_3_ = 240° (blue path 2) and α_3_ = 180° (black path 1) with α_1_ = 0°. (**b**) Minimal condition number vs final angle, or equivalently path length. The condition number varies by less than one order of magnitude for final angles α_3_ > 240°, which indicates that a full revolution of Ma1 might not be required.

We focussed on a helical path with one revolution, *α*_3_ = 360° and the height varying from *z*(*α*_1_) = −0.15 m to *z*(*α*_3_) = 0.15 m (i.e. total array height). Figure 2a illustrates three different 3D paths with linear height variation, *α*_2_ = 180° (red path 2) and non-linear height variations *α*_2_ = 100° (black path 3) and *α*_2_ = 240° (blue path 1). We also evaluated different helical path lengths (Fig. 3a) by selecting *α*_3_ = 180° (black path 1), *α*_3_ = 240° (blue path 2) and *α*_2_ = 360° (red path 3). In each figure, the small line segments indicate the spatial magnetisation vector pointing outwards and perpendicular on the path at each encoding step (see insets). The quality of the reconstructed image was evaluated using the mean squared deviation from the digital phantom.

## Results

For all magnet configurations considered, the rank of the encoding matrix varied little. Here we present the results for encoding matrix condition number only.

### Configurations with one encoding magnet

Figure 2c shows the condition number of the encoding matrix versus the encoding magnet Ma1 orientation, described by the azimuthal angle ϕ and polar angle θ (see Fig. 2a), as a grey scale surface plot. Each point represents one encoding condition number for one measurement with a fixed magnet orientation with θ varying from −90° to 90° and ϕ from 0° to 360° in 5° steps. The rotation angle *α* ranges from *α*_1_ = 0° to *α*_3_ = 360°. Three values for *α*_2_ were selected: 180° (Fig. 2c, top), 100° (Fig. 2c, middle) and 240° (Fig. 2c, bottom). For *α*_2_ = 180° *z*(α) is a linear function of *α*, otherwise *z*(α) varies quadratically (Fig. 2b). In all cases, a broad region of lower condition number is present around θ = 0 and ϕ = 0 i.e. with the magnetization oriented perpendicular to the path. Figure 2d depicts the dependence of the lowest condition number of each configuration on *α*_2_, with path parameters *α*_1_ = 0° and *α*_3_ = 360°. The condition number is lowest for *α*_2_ = 180°.

Figure 3 shows the effect of varying path length on the condition number for one encoding magnet. Three paths length are shown in Fig. 3a with *α*_3_ = 180° (black path 1), *α*_3_ = 240° (blue path 2) and *α*_3_ = 360° (red path 3) with linear height variation of the helical path. Figure 3b illustrates that the condition number significantly increases as path length decreases but varies by less than one order of magnitude for α_3_ between 240° and 360°. This may enable faster encoding without compromising efficiency.

**Figure 4:**
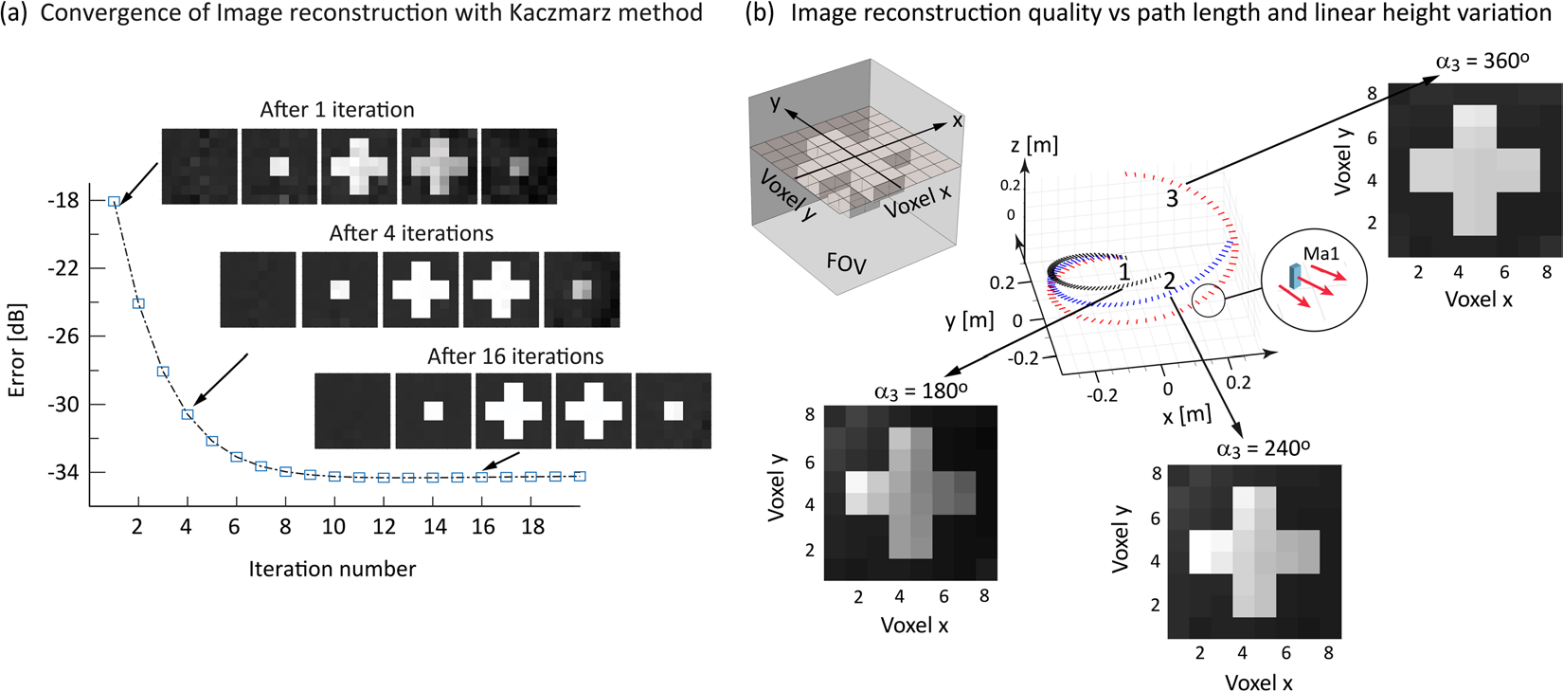
Image reconstruction with one encoding magnet Ma1. (**a**) Calculated error for image reconstruction with an iterative Kaczmarz-based method. The images show 5 cross sections (see text) of the 3D sample (see Fig. 1c) after 1, 4 and 16 iterations. Image convergence occur after about 8 iterations. (**b**) Image quality dependence on path length for α_3_ = 180° (black), α_3_ = 240° (blue) and α_3_ = 360° (red). Images are shown for each path at one cross section through the sample (z = 0, see inset) after 10 iterations with standard deviations calculated for α_3_ = 180° (0.0231), α_2_ = 240° (0.0221) and α_2_ = 360° (0.0200).

Figure 4a shows 2D transverse cross section images at *z* = 0.06 m, 0.045 m 0.015 m, −0.015 m and −0.045 m achieved with a single encoding magnet *Ma*_1_ and with path parameters *α*_1_ = 0°, *α*_2_ = 120° and *α*_3_ = 240° (Fig. 4b, blue path 2), reconstructed with the Kaczmarz method. Results for different iteration numbers are shown with image quality improves rapidly within the first few iterations and convergence occurs within 5–8 iterations. For further evaluation of the quality of reconstructed images we arbitrarily selected 10 iterations as the comparator against the digital phantom. The effect of path length on spatial encoding and image reconstruction quality is illustrated in Fig. 4b, which shows images in the *xy*-plane at *z* = 0 m. Greater path length results in lower standard deviation between reconstructed and phantom images: standard deviation = 0.0231 for *α*_3_ = 180°, 0.0221 for *α*_3_ = 240° and 0.0200 for *α*_3_ = 360°.

### Optimization and image reconstruction with two encoding magnet

**Figure 5:**
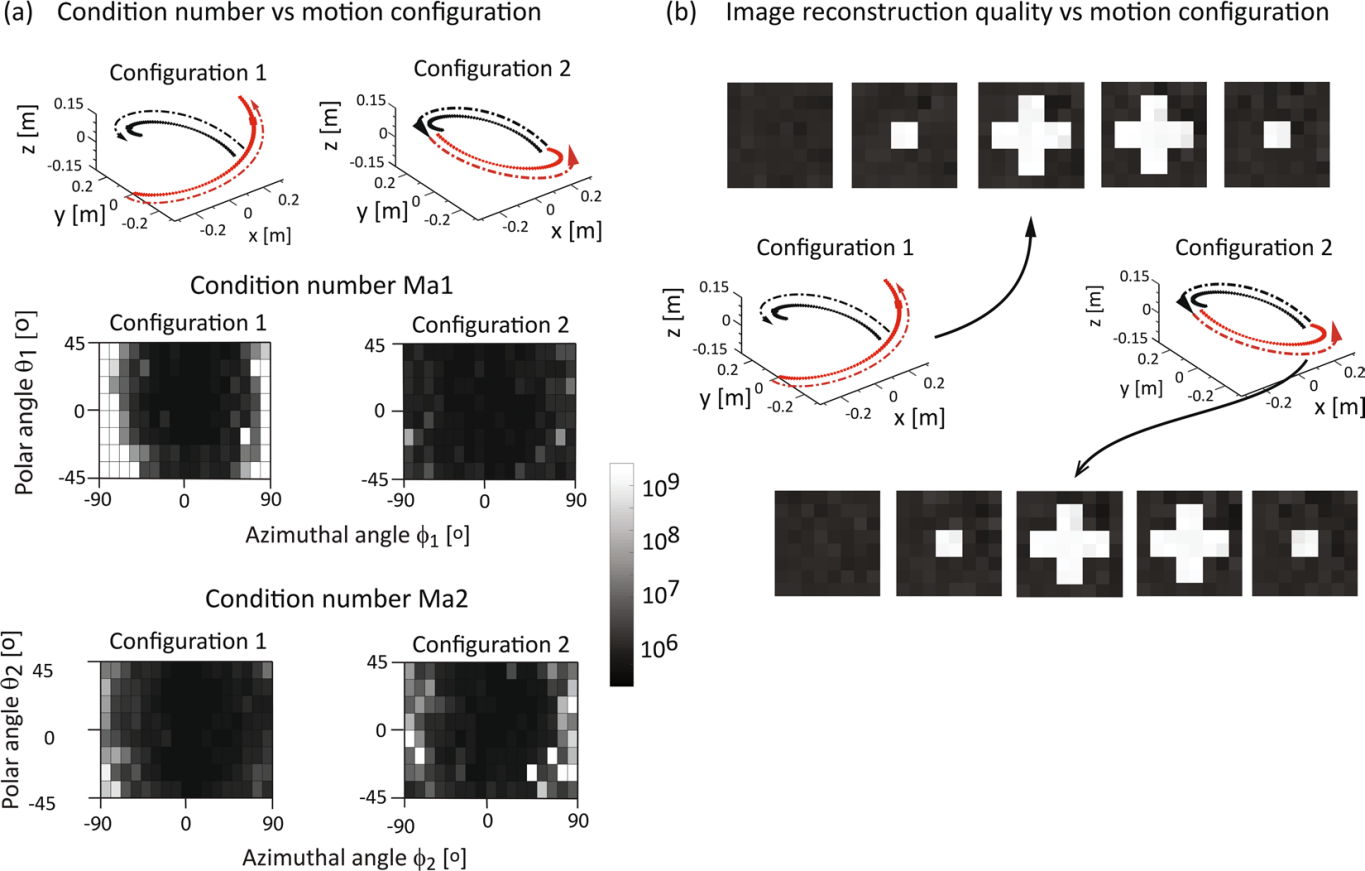
Condition number vs magnet orientations and image reconstruction with Ma_1_ and Ma_2_. (**a**) The paths and the arrows indicate the magnet motion with configuration 1 (left column) and configuration 2 (right) column. At each encoding step the magnets are opposite to each other (xy-plane projection). The condition number distribution is shown for Ma_1_ assuming optimal orientation of Ma_2_ and vice versa. Like for one encoding magnet, the optimal orientations are θ ≈ 0° and ϕ ≈ 0° for both encoding magnets. (**b**) Image reconstruction for Ma_1_ (black) and Ma_2_ (red) indicated by the arrows for two configurations shown after 10 iterations. The cross section locations correspond to Fig. 4. The standard deviations are 0.0254 (configuration 1) and 0.0287 (configuration 2).

We next considered the case of two identical magnets moving along two path configurations as shown in Fig. 5. Configurations were examined in which magnet *Ma*_1_ moves counter clockwise from the bottom to the top (Fig. 5a, black curves and arrows) and magnet Ma_2_ moves counter clockwise from the bottom to the top (Configuration 1, Fig. 5a, red curve and arrow) or from top to bottom (Configuration 2, Fig. 5a, red curve and arrow). The magnets were separated by 180° at all times to reduce image inhomogeneity. The combined path lengths of both magnets was chosen to equal the circumference of *array D*.

The polar and azimuthal angles of the encoding magnets *Ma*_1_ and *Ma*_2_, were independently varied to determine the minimal condition number and optimal orientation. Figure 5a shows the condition numbers for *Ma*_1_ for different combinations of ϕ_1_ and θ_1_ keeping ϕ_2_ and θ_2_ for *Ma*_2_ at their optimum (left panel shows results for Configuration 1 and right panel shows results for Configuration 2) and the condition numbers for *Ma*_2_ for different combinations of ϕ_2_ and θ_2_ keeping ϕ_1_ and θ_1_ for *Ma*_1_ at their optimum for each of the corresponding configurations. Optimal orientations angle for two magnets are perpendicular to the magnet path (ϕ_1_°^pt^ and ϕ_2_°^pt^ ~ 0°) and parallel to the xy-plane (θ_1_°^pt^ and θ_2_°^pt^ ~ 0°). The reconstructed images for each configuration are shown in Fig. 5b. The standard deviations for configurations 1 and 2 were 0.0254 and 0.0287 respectively; image quality was higher in the former.

## Discussion

We introduce a novel 3D spatial encoding method using dynamic SPMAs for ULF-MRI. We developed an in-house simulation method to determine optimal magnet orientations and locations for prescribed path parameters depending on the instrument design. Our approach calculates analytically the discrete magnetic field distribution within the field of view generated by localized magnetic dipoles. The dipole approximation is applicable and accurate since the encoding magnet sizes are assumed to be much smaller compared to the characteristic distances (see Fig. 1). Our approach allows faster calculation since only practical feasible solutions are considered for specific construction designs. We describe an encoding array designs with one or two magnets for an ULF-MRI instrument we have developed, using simple helical magnet motions. Although only spiral paths with equidistant stopping points along a cylindrical surface were considered, the semi-analytical method can be readily extended to include any number of magnets moving along any prescribed paths.

MATLAB’s inbuilt functions *rank* and *cond* were respectively employed to calculate the rank and condition number, of the resulting encoding matrix. The maximum rank equals the number of encoding field configurations, *q*, times signal acquisition number *N* per encoding field and determines the total voxel number.

We applied the Kaczmarz method, an iterative algorithm for solving the linear equation 3. Based on the results summarized in Fig. 4a we assumed 10 iterations until image convergence before attempting image comparison using the standard deviation from the phantom image. This allows us to compare the resolving power of the different encoding fields and therefore the reconstructed image quality.

Our simulations predict that with a single encoding magnet moving around the sample on a linear helical path 3D images can be acquired without moving the sample or applying additional encoding RF pulses like Bloch-Siebert spatial encoding (BS-SET) or transient array spatial encoding (TRASE)^16^. For the design studied, we found lowest condition numbers were achieved when the height variation *z*(*α*) was a linear function of *α*. This is attributed to the low helical path slopes for the non-linear height variation near the bottom (black curve 1, *α*_2_ = 100°) and the top (blue curve 3, *α*_2_ = 240°; see Fig. 2b) which lead to lower variation in the encoding field along the z-axis and hence increased linear dependencies and higher condition numbers.

Shortening the path length with constant height variation increased condition number and reduced the quality of the reconstructed image (Fig. 4b). This is not unexpected because the step size decreases with reduced path length if the number of voxels is unchanged, leading to increase linear dependence between encoding field configurations. Additionally, due to the drop in field strength with distance, variation in Larmor frequency in the sample is smaller at locations furthest from the magnetic dipole. Image quality is degraded if the encoding magnet does not fully revolve about the sample (see Fig. 4b, for *α*_3_ = 180° and *α*_3_ = 240°). Increasing path lengths with one encoding magnet to enhance image quality increases acquisition time and may require more complex mechanical motion control. This can be alleviated by introducing multiple encoding magnets, each controlled independently.

For the configurations considered, the optimal magnet orientations were perpendicular to both the motion path and the cylindrical surface of Array D. This can be attributed to the magnetic field distribution of a magnetic dipole which has a larger field gradient along its magnetization direction^26^. This result might be expected because of the cylindrical structure of the instrument but cannot be generalized to further simplify the optimization process without more detailed analysis, which is beyond the scope of this study.

For all cases considered, the distribution of encoding matrix condition number (Figs 2c and 5a) is relatively flat in broad regions around the minimum values. This indicates a high manufacturing tolerance for the construction of the encoding array including encoding magnet alignments and helical paths. Changes in magnitude and orientation of the magnetic field or in the mechanical device can be taken into account in the encoding matrix during the calibration of the instrument. External static magnetic fields or transient effects like temperature drifts or mechanical vibrations may also be corrected using additional sensors^6,16,27^ or software gradiometry to remove external fields from the signal^28^.

An additional potential advantage of permanent magnet encoding arrays is the ability to control 3D field variations to further enhance image resolution locally. This has been used in Parallel Acquisition Technique with LOCalised gradients (PATLOC) to better match the imaging geometry of interest in high field MRI^29^. However, the coil arrangement offers local image enhancements in 2D at fixed locations only. In principle, a flexible and modular permanent magnet encoding arrangement allows resolution to be enhanced at any location within the sample by spatially varying the paths and magnet orientations to control magnitude and spatial encoding field distribution.

## Conclusion

The spatial non-linear encoding design, based on moving magnets, presented in this paper is substantially different from conventional coil-based linear gradient devices reported in the literature to date. We show in principle that a single encoding magnet revolving around a sample suffices for imaging with back projection. Mechanical magnet motions and adjustments are not time critical since they are confined during the non-measurement period during pre-polarization. With the restriction of spatially linear magnetic fields lifted, the potential advantages of permanent magnet arrays for ULF-MRI operation can be realized. These include 3D imaging of a stationary sample, slice selection and local image resolution enhancement.
